# US Tuberculosis Rates among Persons Born Outside the United States Compared with Rates in Their Countries of Birth, 2012–2016^1^

**DOI:** 10.3201/eid2603.190974

**Published:** 2020-03

**Authors:** Clarisse A. Tsang, Adam J. Langer, J. Steve Kammerer, Thomas R. Navin

**Affiliations:** Centers for Disease Control and Prevention, Atlanta, Georgia, USA

**Keywords:** Tuberculosis, latent tuberculosis, emigration and immigration, epidemiology, incidence, tuberculosis and other mycobacteria, country of birth, United States, bacteria

## Abstract

The US Centers for Disease Control and Prevention recommends screening populations at increased risk for tuberculosis (TB), including persons born in countries with high TB rates. This approach assumes that TB risk for expatriates living in the United States is representative of TB risk in their countries of birth. We compared US TB rates by country of birth with corresponding country rates by calculating incidence rate ratios (IRRs) (World Health Organization rate/US rate). The median IRR was 5.4. The median IRR was 0.5 for persons who received a TB diagnosis <1 year after US entry, 4.9 at 1 to <10 years, and 10.0 at >10 years. Our analysis suggests that World Health Organization TB rates are not representative of TB risk among expatriates in the United States and that TB testing prioritization in the United States might better be based on US rates by country of birth and years in the United States.

In the United States, 9,272–9,940 cases of tuberculosis (TB) were reported annually during 2012–2016; incidence rate was 2.9–3.2 cases/100,000 population (*1*). Most cases occurred among non–US-born persons and were attributed to progression of remotely acquired latent TB infection (LTBI) rather than recent transmission within the preceding 2 years (*2*). US law requires non–US-born persons seeking lawful permanent residency in the United States and refugees resettling in the United States to undergo a medical examination, including screening for TB. These examinations can be done by panel physicians in other countries before the immigrant or refugee arrives in the United States, or they can be done by civil surgeons in the United States for persons with temporary visas who are seeking lawful permanent residency (*3*,*4*). This TB screening requirement is intended to identify otherwise undiagnosed TB among non–US-born persons seeking lawful permanent residency, although it would not identify TB among the substantial proportion of non–US-born residents who are in the United States on temporary visas or who are undocumented. Of note, these required examinations would identify any cases of TB among examinees, but testing of adults for LTBI is not required. Although recent reliable data on visa status of US TB patients are not available, Davidow et al. determined that among non–US-born TB patients reported during 2005–2006, most (61%) were either permanent residents or naturalized citizens who would have been screened for TB either before departure or during visa status change in the United States, whereas 13% were temporary visa holders (student, work, or exchange visas) for whom TB screening is not mandated and 25% were undocumented (*5*).

The US Centers for Disease Control and Prevention (CDC) and the US Preventive Services Task Force recommend that healthcare providers and public health departments offer testing for TB infection in populations at increased risk for exposure to TB or for having TB infection progress to TB. This recommendation encompasses persons who were born in or frequently travel to countries with high numbers of TB cases among their expatriates living in the United States, including Mexico, the Philippines, Vietnam, India, China, Haiti, and Guatemala, as well as other countries where rates of TB are high (*6*). What constitutes high TB rates or how these rates should be calculated is not specified. One option is to use TB incidence rates in countries as reported by the World Health Organization (WHO; hereafter referred to as WHO rates); however, using WHO rates assumes that TB rates in a given country reflect TB rates among persons from that country in the United States (hereafter referred to as US rates). We compared WHO rates with US rates for all countries for which data were available. Our hypothesis was that using US rates by country of birth would provide better data than using WHO rates, which is the current US Preventive Services Task Force recommendation for establishing which countries have a high burden of TB (*7*).

## Materials and Methods

We analyzed data from the US National TB Surveillance System (NTSS) for TB cases reported during 2012–2016 for persons born outside the United States. To minimize the effects of yearly fluctuations in rate, we selected a 5-year period; at the time of this analysis, the most recent data were from 2016. NTSS data are compiled from reports of TB cases submitted electronically to CDC by the 50 states and the District of Columbia. Reports include the patient’s self-reported country of birth, approximate date of arrival in the United States, other demographic information (e.g., date of birth, sex, race/ethnicity), and clinical information (e.g., site of TB disease and laboratory results). For this analysis, persons born outside the United States included persons born in the US territories (American Samoa, Commonwealth of the Northern Mariana Islands, Guam, Puerto Rico, and US Virgin Islands). Because persons born in the US territories are eligible for US citizenship, they do not report a month or year of arrival in the United States. Persons from the US sovereign freely associated states of the Federated States of Micronesia, the Republic of the Marshall Islands, and the Republic of Palau are classified as born outside the United States but do not require a visa to visit or relocate to the United States. In this way, they are similar to persons born in the US territories. NTSS does not collect travel history or countries of residence other than among pediatric patients (<15 years of age).

To calculate TB rates in the United States by patient’s country of birth, we obtained TB case counts by country of birth reported to NTSS during 2012–2016 as well as US population estimates by country of birth from the US Census Bureau, American Community Survey (ACS), Public Use Microdata Sample data, 2012–2016 multiyear file (*8*). ACS is an annual survey of ≈3.5 million US households and includes reported country of birth and year of arrival in the United States. We also calculated rates by years spent in the United States before TB diagnosis (<1 year, 1 to <10 years, and ≥10 years). Case rates were calculated as the number of cases per 100,000 population.

In the ACS, country of birth is based on self-report and coded as a country or region. Persons born outside the United States are asked to report country of birth according to current international boundaries. If there are <10,000 persons from a particular country of birth in the United States, the ACS does not provide population estimates from that particular country but groups the country into a region. The ACS does not provide individual population estimates for North and South Korea but provides a population estimate for Korea as a whole, encompassing the populations of South and North Korea. The ACS also does not provide a population estimate for the state of Palestine. For TB case data for persons born in countries where the ACS does not provide individual country population estimates, we aggregated these cases into regions by using the same regions categorized by the ACS (*8*) and applied the TB rate calculated for the region to all countries included in that region. If a country of birth associated with a case reported to NTSS was not provided as a country of birth or categorized within a region by the ACS, then a rate was not calculated. We compared our US TB rate calculations by country of birth (US rates) with country TB rates published by WHO for 2014 (WHO rates), which was the midpoint year between 2012 and 2016 (*9*).

To compare the WHO and the US rate estimates, we calculated incidence rate ratios (IRRs) for each country of birth. IRRs >1.0 indicated a higher WHO rate than the corresponding US rate. We used 95% CIs from 2014 WHO incidence rates to calculate 95% CIs for each individual country/region-specific IRR by dividing the WHO lower CI limit by the US rate and the WHO upper CI limit by the US rate. We also compared WHO rates with US rates by years since US arrival by calculating median IRRs by year. We used SAS version 9.4 (https://www.sas.com) to conduct the analysis.

## Results

**Figure 1:**
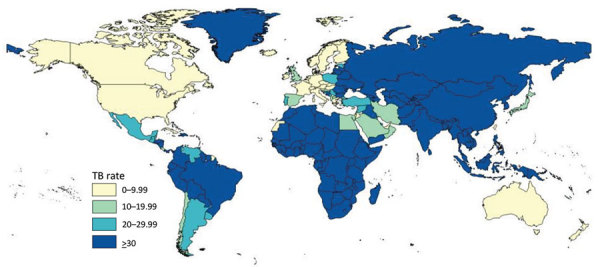
TB rates (per 100,000 population) worldwide, according to World Health Organization reports, 2014. TB, tuberculosis.

**Figure 2:**
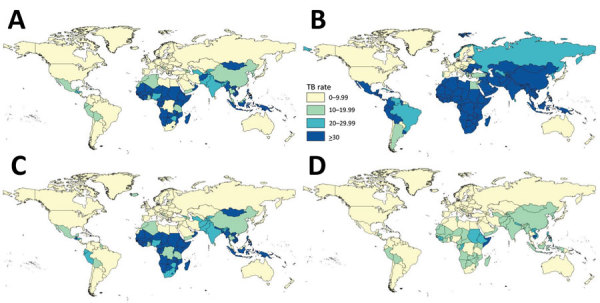
TB rates (per 100,000 population) in the United States, by country of birth and time from US arrival to TB diagnosis, 2012–2016. A) Persons born abroad by their country of birth (note that rates could not be calculated for 1 country); B) persons by their country of birth who lived in the United States <1 year before diagnosis; C) persons by their country of birth who lived in the United States >1 to <10 years before diagnosis; D) persons by their country of birth who lived in the United States >10 years before diagnosis. Note that the US Census Bureau American Community Survey provides only a combined population estimate for Korea; thus, the rate represented for North Korea and South Korea is calculated as a combined rate for Korea. TB, tuberculosis.

During 2012–2016, a total of 47,718 persons in the United States were reported to have TB, of which 32,087 (67.2%) had been born outside the United States. Patients from Mexico, the Philippines, India, Vietnam, and China accounted for 54.0% of persons with reported TB who had been born outside the United States. Rates varied by country and region of birth. US rates were >30 cases/100,000 population for far fewer countries than were WHO rates (Figure 1; Figure 2, panel A). Of the 195 countries in the world, the corresponding US rate was >30 cases/100,000 population for 65 countries, 20–30/100,000 for 13 countries, 10–20/100,000 for 19 countries, and <10/100,000 for 97 countries; the rate could not be calculated for 1 country. Complete data for month and year of arrival in the United States were available for 90% of persons born outside the United States. Rates were generally higher among persons for whom TB was diagnosed <1 year after arrival in the United States than among those who arrived 1 to <10 years before receiving a TB diagnosis (Figure 2, panels B, C) and higher among those for whom TB was diagnosed 1 to <10 years after US arrival than among those who arrived in the United States >10 years before receiving a TB diagnosis (Figure 2, panels C, D). Rates were >30 cases/100,000 population among US residents born in sub-Saharan Africa, South Central Asia, Southeast Asia, Mexico, and parts of Central and South America who received their TB diagnosis <1 year after arrival in the United States. Rates were >30 cases/100,000 population among US residents born in parts of sub-Saharan Africa and Southeast Asia who received their TB diagnosis 1 to <10 years after their arrival in the United States. Rates were >30 cases/100,000 population among only those US residents from the Republic of the Marshall Islands, Somalia, and Cambodia who had received their TB diagnosis >10 years after arrival in the United States. US rates were lower than WHO rates among persons from South America (Figures 1, 2).

Among the 20 countries of birth for which TB case counts in the United States were the highest, the 10 countries for which WHO rates were the highest were the Philippines, Cambodia, Myanmar, Somalia, Pakistan, India, Nigeria, Ethiopia, Haiti, and Laos (range 189–546 cases/100,000 population) (Table 1). In contrast, the 10 areas for which US rates (i.e., among expatriates in the United States) were the highest were Republic of Congo, Republic of the Marshall Islands, Somalia, Bhutan, Myanmar, Nepal, Guinea, Ethiopia, the Federated States of Micronesia, and the countries in the Other Africa region (range 62–150 cases/100,000 population) (Appendix).

**Table 1:** TB rates in the United States, by country of birth, 2012–2016, compared with World Health Organization rates, 2014, for the 20 countries with the highest TB counts in the United States *

COB	Average annual no. cases	Estimated population†	Rate by years since US arrival			Overall US rate by COB	WHO rate (95% CI)‡	IRR (95% CI)§
			<1	1 to <10	>10			
Mexico	1,262.2	11,851,810	103.0	11.7	7.9	10.6	21 (16–27)	2.0 (1.5–2.5)
Philippines	788.4	2,048,557	297.4	42.5	26.8	38.5	54 (304–859)	14.2 (7.9–22.3)
India	537	2,235,594	117.1	24.1	14.1	24.0	223 (136–332)	9.3 (5.7–13.8)
Vietnam	487.2	1,340,215	290.7	46.8	23.8	36.4	140 (111–173)	3.9 (3.1–4.8
China	393.2	1,966,551	56.8	16.7	16.7	20.0	68 (58–78)	3.4 (2.9–3.9)
Guatemala	193.4	929,637	220.9	30.4	8.6	20.8	25 (19–31)	1.2 (0.9–1.5)
Haiti	174.8	661,301	311.9	37.3	13.9	26.4	200 (154–253)	7.6 (5.8–9.6)
Ethiopia	151.8	222,559	623.8	80.4	24.5	68.2	207 (134–295)	3.0 (2.0–4.3)
Honduras	135.8	594,066	231.3	25.2	10.9	22.9	40 (30–50)	1.7 (1.3–2.2)
Myanmar	113.8	129,594	707.3	76.5	15.8	87.8	369 (269–484)	4.2 (3.1–5.5)
El Salvador	107.4	1,330,323	87.4	11.8	4.8	8.1	44 (34–56)	5.5 (4.2–6.9)
Somalia	96.2	85,871	1,033.9	105.6	51.0	112.0	274 (177–391)	2.4 (1.6–3.5)
Nepal	83.4	108,099	439.5	67.6	24.4	77.2	158 (139–178)	2.0 (1.8–2.3)
Peru	82.2	450,546	228.9	28.6	11.3	18.2	121 (93–153)	6.6 (5.1–8.4)
Pakistan	80.8	368,845	178.1	20.4	13.9	21.9	270 (175–386)	12.3 (8.0–17.6)
Cambodia	75.2	161,226	187.4	39.1	36.0	46.6	390 (252–557)	8.4 (5.4–11.9)
Laos	72.4	192,908	96.5	46.6	29.7	37.5	189 (122–270)	5.0 (3.3–7.2)
Ecuador	72.2	439,795	127.8	27.4	9.4	16.4	41 (31–51)	2.5 (1.9–3.1)
Nigeria	68.4	289,679	242.3	28.5	8.1	23.6	219 (143–311)	9.3 (6.1–13.2)
Dominican Republic	66.4	1,064,665	55.2	7.4	4.0	6.2	53 (41–67)	8.5 (6.6–10.7)

*Although South Korea has an average annual number of 97.4 cases, it is not listed in the table because the ACS does not provide a population estimate for South Korea. ACS, US Census Bureau American Community Survey; COB, country of birth; IRR, incidence rate ratio; TB, tuberculosis; US, United States, WHO, World Health Organization.  †ACS Public Use Microdata Sample data, 2012–2016 multiyear file, https://www.census.gov/programs-surveys/acs/data/pums.html. ‡World Health Organization TB burden estimates, https://www.who.int/tb/country/data/download/en/. §Rate in country (WHO rate) divided by overall US rate by COB. An IRR >1.0 indicates that the WHO rate is larger than the US rate.

**Figure 3:**
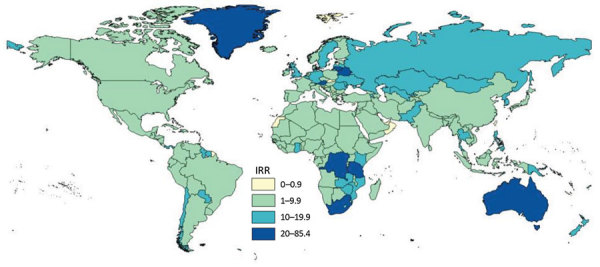
World Health Organization (WHO) versus US TB IRRs. IRR is the rate in country (WHO rate, 2014) divided by the rate by country of birth in the United States (US rate, 2012–2016). IRR >1.0 indicates that the WHO rate is larger than the US rate. IRR, incidence rate ratio; TB, tuberculosis.

Of the 195 countries represented as members of the United Nations (*10*,*11*), rate data for calculation of IRR were available for 189; for 178 (94%) of those, WHO rates were higher than the corresponding US rates (Figure 3). The median IRR was 5.4 (interquartile range [IQR] 2.6–8.7). The country of birth for which IRRs were highest and for which population estimates were not from a region were South Africa, Lithuania, and Belarus. The country of birth for which TB cases were reported and the IRR was notably below average was United Arab Emirates (Appendix).

We calculated median IRRs by years since entry into the United States before TB diagnosis by using the 189 countries. The median IRR was 0.5 (IQR 0.2–1.1) for persons for whom TB was diagnosed <1 year after US arrival, 4.9 (IQR 2.6–9.4) for 1 to <10 years, and 10.0 (IQR 4.9–19.9) for >10 years. Of the 195 countries, the US rate was greater than the WHO rate for 58% of those for whom TB was diagnosed <1 year after US arrival. On average, among persons for whom TB was diagnosed <1 year after US arrival, US rates by country of birth were higher than corresponding WHO rates by country. In contrast, on average among persons for whom TB was diagnosed >1 year after US arrival, US rates by country of birth were lower than corresponding WHO rates by country.

## Discussion

Our analysis showed that, for 2012–2016, the WHO rate (TB incidence rate for a country as reported by WHO) did not consistently equate to the overall US rate (TB incidence in the United States for persons born in the corresponding country). WHO rates, by country, were a median of 5.4 times higher than US rates among persons born in the corresponding countries. The WHO rate for South Africa (820 cases/100,000 population) was most discrepant with the overall US rate for persons born in South Africa (9.6/100,000). Menzies et al. examined the 30 countries with the highest case counts in the United States from 2003 through 2015 and estimated that TB incidence rates in a country of birth were 6.8 times higher than TB rates among persons born in that country and living in the United States (*12*). A previous analysis of 2004 data by Cain et al. demonstrated that US TB rates among non–US-born persons were highest among persons born in sub-Saharan Africa, followed by South Asia, East Asia, and the Pacific (*13*). Another analysis done by Cain et al. showed that annual TB rates for persons in the United States who had been born in Canada, Australia, New Zealand, countries of western Europe, and Japan were very low (<10/100,000 population) (*14*). Our results are consistent with the previous findings that TB rates are highest among US persons from sub-Saharan Africa, Asia, and the Pacific Islands (Figure 2).

Current US TB screening guidelines recommend TB testing for persons born in countries where TB rates are high (*6*). Although what constitutes high TB rates is not specified universally, several resources use WHO rates to dictate screening practices (*15*,*16*). Our data show that WHO rates differ from US rates. WHO TB rates by country were a median of 5.4 times higher than the TB rate in the United States for persons from the corresponding country of birth (Table 1). Time since arrival in the United States also plays a role in TB rates. On average, among persons for whom TB was diagnosed <1 year after US arrival, US rates for the country of birth were higher than corresponding WHO rates (median IRR 0.5) (Table 2). This finding is consistent with previous findings. Cain et al. showed that TB rates for non–US-born persons were highest for those who had been in the United States <1 year before TB diagnosis (121.0 cases/100,000 population) and lowest for those who had been in the United States >5 years before TB diagnosis (11.9/100,000 population) (*13*). Similar to our analysis, that analysis also showed that for most non–US-born persons, rates of TB among those who had been in the United States <1 year were higher than rates for their respective countries of birth (*13*). High rates of TB among persons who had been in the United States for <1 year could be attributed in part to selection bias resulting from immigration-related TB screening. Walter et al. demonstrated that 85% of TB cases diagnosed for immigrants from the Philippines in California within 1 year of their preimmigration examination in the Philippines were attributed to imported TB (i.e., prevalent TB at the time of arrival in the United States) (*17*). Cain et al. demonstrated that even for non–US-born persons who had lived in the United States for >20 years, annual TB case rates were >10 cases/100,000 population (*14*). This finding is further supported by the Walter et al. study, in which the rate of LTBI reactivation among immigrants with negative preimmigration examination results (no evidence of TB detected by physical examination, radiography, sputum culture, or sputum microscopy) was 32 cases/100,000 population/year within 9 years of US entry (*17*). Thus, when addressing US rates, it is crucial to examine TB rate by years since arrival in the United States. Despite TB rates being higher among persons who had been in the United States for <1 year than rates reported by WHO for the same country, almost half of non–US-born persons with TB are among those who have lived in the United States for >10 years (*18*,*19*). Other age-period-cohort effects might also be useful when comparing US and WHO rates by country of birth. A previous study by Iqbal et al. showed that TB rates decreased as members of a birth cohort aged but were higher among adolescents and young adults (*20*). Another finding from that study was that, among non–US-born persons during 1996–2016, TB rates among black persons were highest for those <45 years of age, but rates among Asians/Pacific Islanders were highest for those >45 years of age (*20*). Of note, a study in Canada showed that persons born in countries where TB incidence was lower had arrived in Canada in earlier years than those from countries where TB incidence was higher (*21*).

**Table 2:** World Health Organization versus US TB IRRs, by years since entry into the United States before TB diagnosis*

Year(s) since entry into the United States	Median IRR†
<1	0.5
1–4	4.0
5–9	6.7
10–14	6.9
15–19	11.0
20–24	11.1
25–29	10.9
30–34	7.2
35–39	7.1
40–44	9.3
45–49	5.5
50–54	6.7
>55	4.9

*COB, country of birth; IRR, incidence rate ratio; TB, tuberculosis; WHO, World Health Organization. †IRR is the rate in country (WHO rate, 2014) divided by the rate by COB by years since entry into the United States (US rate, 2012–2016). An IRR >1.0 indicates that the WHO rate is larger than the US rate. Median IRR for each year since entry into the United States category was calculated for the 195 countries defined by the United Nations Member State (https://www.un.org/en/member-states/index.html) and Non–Member States (https://www.un.org/en/sections/member-states/non-member-states/index.html) lists. A WHO rate and a nonzero US rate were available for only 189 of these countries.

A strength of examining rates of TB in the United States by country of birth is that US TB surveillance data are relatively complete. Winston et al. conducted an in-depth investigation of 11 US reporting areas that together reported 5,436 TB cases in 2008 and 2009 and did not find a single unreported case (*22*). Furthermore, the US Census Bureau ACS is the largest US household survey with reliable data on demographic, social, economic, and housing measures. Because completion of ACS is mandatory, levels of nonresponse are low (*23*). When examining global data, WHO does not measure TB incidence at national levels because of high costs and challenging logistics. Methods currently used by WHO to estimate TB incidence are based on case notification data combined with expert opinion about case-detection gaps, TB prevalence surveys, notifications in high-income countries adjusted for underreporting and underdiagnosis, and inventory studies and capture–recapture analyses (*24*). WHO uses population estimates from the United Nations, where recent country-level population data may not always be available; thus, standard demographic adjustments are made (*25*). Because US surveillance of TB reporting is complete and the ACS provides detailed population data, we believe that US rates by country of birth are more appropriate than WHO rates for prioritizing TB testing in the United States. In addition, using rates by country of birth might be helpful for local TB programs interested in TB targeted testing within their own jurisdictions (*26*,*27*). Using rates by country of birth, Readhead et al. demonstrated that the highest TB rates in Los Angeles County, California, were among persons born in Myanmar, Ethiopia, and Indonesia (*28*).

Our study has limitations. Incidence rate calculations are based on estimated population denominators. NTSS and the ACS rely on self-report for country of birth. Because numerous foreign country boundaries have changed in the past century, some persons may report country of birth in terms of boundaries that existed at the time of their birth or emigration or in accordance with their own national preference. We used the midpoint WHO year of 2014 to compare with the ACS population denominators from 2012–2016. In addition, the method used by WHO for calculating country rates differs from our approach for calculating rates by country of birth in the United States.

Most TB cases in the United States are in persons born outside the United States (*1*), and most cases are attributed to progression of remotely acquired TB infection. Statistical modeling has shown that the most effective intervention for reducing the overall US TB rate is treatment of LTBI (*29*); rate reduction is predicted to be greater among non–US-born than among US-born persons (*30*). However, available resources for TB prevention programs remain limited, which highlights the need to ensure that TB testing programs are as cost-effective as possible. Tasillo et al. demonstrated the cost-effectiveness of testing and treating LTBI among non–US-born persons (*31*). 

Our analysis emphasizes the value of focusing on country of birth and length of time in the United States to guide how to best expand LTBI testing and treatment. Other factors, such as underlying conditions and socioeconomic disparities, could play a role in TB risk (*32*). Accordingly, populations should be prioritized for TB testing according to their relative risk of being TB infected or having TB develop. This prioritization is especially relevant in a low-incidence country like the United States, where careful consideration must be made with regard to LTBI screening to ensure that the benefits outweigh the harm (*33*). Historically, risk for TB infection in non–US-born populations has been based on WHO TB incidence rates (*15*,*16*); however, our analysis shows the differences between WHO rates by country and US rates by country of birth. We believe that US rates are more relevant than WHO rates for TB screening in the United States because expatriates living in the United States differ from the population of their country of birth. Our analysis, in combination with the accuracy and completeness of NTSS and ACS data compared with reported WHO TB incidence rates (*22*), demonstrates that country of birth–specific US rates provide a better method for prioritizing populations for testing in the United States. A next step would be developing a strategy that uses US country-of-birth rates and length of time in the United States to designate cutoff points to prioritize testing for TB infection among persons born outside the United States.

Appendix Tuberculosis rates by country of birth, United States, 2012–2016 compared with World Health Organization rates, 2014.
